# Enzymes Drive Glutathione Shunt to Explain Oxidative State Using an In-Parallel Multi-Omic Method

**DOI:** 10.3390/ijms26083632

**Published:** 2025-04-11

**Authors:** Valerie C. Wasinger, Sonia Bustamante, Nashwa Najib, Ashish Diwan, Tharusha Jayasena, Nahian S. Chowdhury, Julia Beretov, Siobhan Schabrun

**Affiliations:** 1 Bioanalytical Mass Spectrometry Facility, Mark Wainwright Analytical Centre, University of New South Wales, Sydney, NSW 2052, Australia; 2 Department of Orthopaedic Surgery, St. George and Sutherland Clinical Campuses, School of Clinical Medicine, University of New South Wales, Sydney, NSW 2052, Australia; 3 Centre for Healthy Brain Ageing (CHeBA), Discipline of Psychiatry & Mental Health, School of Clinical Medicine, Faculty of Health & Medicine, University of New South Wales, Sydney, NSW 2052, Australia; 4 Center for Pain IMPACT, Neuroscience Research Australia, Sydney, NSW 2031, Australia; 5 School of Psychology, University of New South Wales, Sydney, NSW 2052, Australia; 6 St. George and Sutherland Clinical Campuses, School of Clinical Medicine, University of New South Wales, Sydney, NSW 2052, Australia; 7 Cancer Care Centre, St. George Hospital, Kogarah, NSW 2217, Australia; 8 Department of Anatomical Pathology, NSW Health Pathology, St. George Hospital, Kogarah, NSW 2217, Australia; 9 Gray Centre for Mobility & Activity, Parkwood Institute, St. Joseph’s Healthcare, London, ON N6C 0A7, Canada; 10 School of Physical Therapy, University of Western Ontario, London, ON N6A 3K7, Canada

**Keywords:** glutathione shunt, liquid biopsy, GSH, GSSG, SLCA7A1, GPx, IBD, biomarker

## Abstract

The glutathione shunt is one of the most important contributors to the cellular redox state, with implications across cancer, chronic diseases, diseases of ageing, and autoimmune diseases, including inflammatory bowel disease (IBD). Traditionally, the redox state is gauged by the ratio of the surrogate metabolites GSH and GSSG. However, this presents methodological challenges and offers a constrained illustration of metabolites without a systems-level understanding of redox dynamics, failing to elucidate variations across an entire biochemical network. Targeted proteomics can fill this void. Here, we describe an in-parallel metabolomic and proteomic targeted method to encompass measurements directly related to the shunt. Samples are simultaneously prepared to extract the substrate building blocks, cysteine, cystine, methionine, glutamic acid, and kynurenine; and the proteins, SLC7A11 (xCT), Glutamate Cysteine Ligase (GSH1), Glutathione Synthetase (GSH2), Glutathione Peroxidase (GPx), and Glutathione Reductase (GSHR) for targeted mass spectrometry. We demonstrate the method by targeted analysis of proteins in plasma, serum, nasal swab, and saliva and apply the multi-omic method to assess changes in the glutathione shunt in the serum of patients diagnosed with IBD. This allows for a broader narrative to establish context at which the glutathione shunt is operating.

## 1. Introduction

Mitochondrial stress leads to the disruption of energy metabolism and inflammatory cues that are a hallmark of many pathological conditions including inflammatory bowel disease [1]. One of these cues relates to redox potential. The glutathione shunt is a crucial mechanism that generates the energy currency NADPH and uses reduced glutathione (rGSH) to balance the redox state. The redox metabolites in the shunt (GSH/GSSG) have become key cellular health indicators [2,3,4]. GSH is the most abundant anti-oxidant nucleophile intracellularly able to modulate redox processes, membrane integrity, with a role in ferroptosis, and the detoxification of xenobiotics [5,6,7]. The biosynthesis of GSH results from a coordinated ATP-dependent two-step enzymatic reaction which first links the amino acids cysteine to glutamate via γ-Glutamyl Cysteine Synthetase (GSH1), followed by the addition of glycine via Glutathione Synthetase (GSH2). GSH primarily exists in the cytosol in its reduced form with two molecules of GSH under oxidative conditions generating glutathione disulfide (GSSG), the oxidised form.

**Figure 1:**
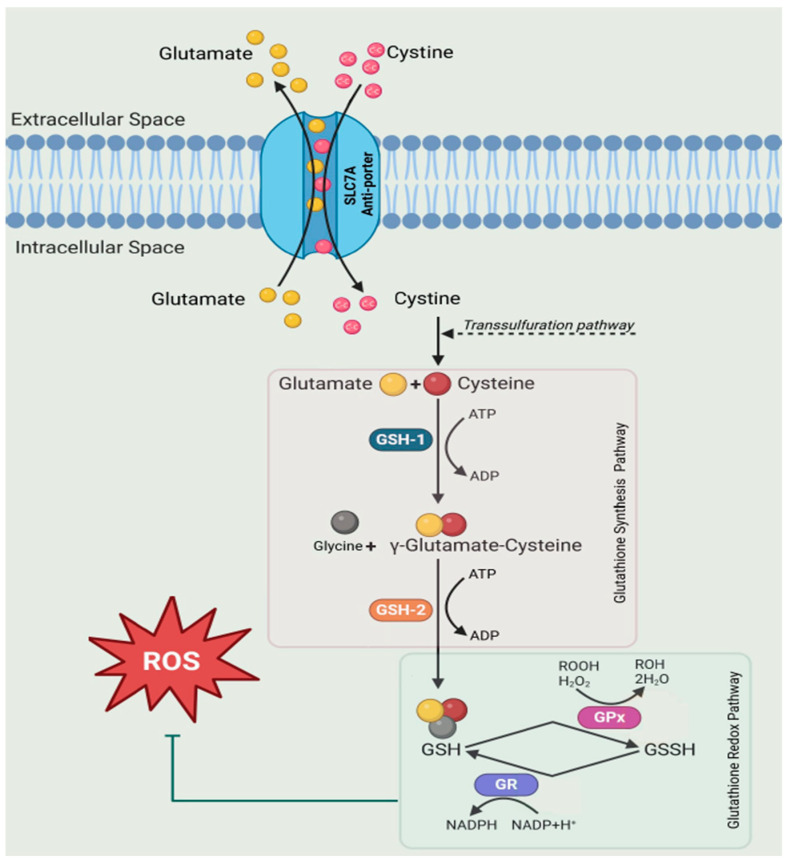
GSH exists as a tri-amino acid combination of glycine, cysteine, and glutamine, with a readily oxidisable thiol group. Increased synthesis and turnover of glutathione as well as an increase in NADPH consumption from the re-generation of the oxidised form (GSSG) supports the antioxidant defence system. In Eukaryotes, GSH1 catalyses the formation of γ-GC, while GSH2 conjugates glycine to form GSH [9]. The antiporter SLC7A transports cystine, cysteine, glutamate, and kynurenine to restore redox homeostasis.

The protein enzymes directly responsible for interconversion between reduced and oxidised glutathione are Glutathione Peroxidase (GPx) and Glutathione Reductase (GSHR), with GPx3 being the predominant peroxidase in serum [8]. These enzymes play a fundamental role in the function of GSH as a redox regulator [3]. Changes in the abundance of these enzymes have the potential to be markers of oxidative stress (Figure 1). This process prevents hydrogen peroxide, lipid hydroperoxides, and organic hydroperoxides, which contribute to membrane permeabilisation, apoptosis, and potentially cell death [5].

The maintenance of redox to control reactive oxygen species (ROS) supports immune cell functions such as T-cells, macrophages, and dendritic cells. The measure of an increased GSH/GSSG ratio is important for neutralising ROS produced in aerobic mitochondrial respiration as a by-product of the electron transport chain [10]. In regulated amounts, ROS can activate transcription, gene expression, and cellular differentiation/proliferation. However, excessive ROS interfere with the mitochondrial membrane potential and the electron transport chain and can cause cell death. This feature is exploited by immune cells to control infection. GPx transforms their by-product hydrogen peroxide to consume rGSH and protect against cellular damage. We propose that the ratio GPx/GSHR may also reflect cellular oxidative stress, with changes in the abundance of the protein ratio an immune trigger signalling the infiltration of cytokines and other immune mediators.

Classical measurements of GSH and/or GSSG have been achieved for decades by: derivatisation in tissue extracts [11,12], fluorimetry [13], HPLC in the ocular lens [14], HPLC-UV in plasma [15], ion-exchange and mass spectrometry on red blood cells in whole blood [16]; and are a powerful measure of cell health. There are well documented methodological challenges recorded across many studies [7,17,18] which have been comprehensively reviewed by others [17,19,20,21]. The challenges surrounding the use of metabolites as a surrogate for cellular oxidative state involve their rapid conversion and degradation. Artificial oxidation of the sulfhydryl groups during sample preparation and indirect measures of GSSG using an –SH masking agent can lead to discrepancies in measures of thiol redox balance [21]. Furthermore, any colourimetric or fluorometric based assays rely on an indirect measure of a derivative as a proxy for GSH levels [4]. Proteins are generally more stable, and as the functional molecules in a biological system, they can be mechanistically more reflective of stress processes. Many methods also require cellular extraction of these metabolites, as GSH is intracellularly assembled and the passive uptake of therapeutically administered GSH is limited due to a prohibitive concentration gradient between intra- and extra-cellular environments [22]. GSH is generated intracellularly, and the proteolytic degradation of GSH and GSSG by γ-Glutamyl Transferase and the excellent thiol scavenging ability result in rapid changes in the metabolite status during sampling and storage which can alter the redox state. Treating samples early with reducing agents can limit this; however, the focus on rGSH rather than total GSH in the literature reflects this challenge [23,24,25,26]. Quantifying the protein enzymes can alleviate this dilemma as the response time to the alteration of enzyme expression is longer (minutes to hours) than the conversion of the metabolites (seconds to minutes). Additionally, the redox potential of a cell and cellular health is both context dependent and multifaceted. Oxidative stress results from excess oxidants (metabolites and free radicals) and/or a dysfunctional anti-oxidant defence system (involving the enzymes) contributing to inflammation. Promiscuity in this system also allows for a number of amino acids to act as an anti-oxidant and each compound with a free –SH moiety to contribute to measures of rGSH [27,28]. There are multiple pinch points in this network, often leading to reported heterogeneity because metabolites, building blocks, and enzymes have not been fully accounted for in the same patient samples at the same time point. Essential understanding of the context in which the shunt operates is dictated by reservoirs of the building blocks and availability of the intra- and extra-cellular resources. Substrates for the shunt include cystine, homo cysteine, glutamate, and glycine, and are ‘re-claimed’ as required upstream, becoming a potential bottleneck in rGSH production [29]. For example, amino acid starvation triggers the integrated stress response (ISR) and, via ATF4, inhibits the enzyme GPx [28,30], thus preventing glutathione dependent detoxification, while limiting the transport of cystine results in cysteine starvation [8,28], and the rate limiting precursor for glutathione decreases GSH with a concomitant increase in the expression of GSHR to activate ROS scavenging [28]. A secondary example is provided by looking more closely at the involvement of the tryptophan pathway metabolism which plays into the modulation of the shunt via the IDO negative cell import of kynurenine through a shared cystine/glutamate anti-porter SLC7A11 (or xCT) and the generation of the kynurenine downstream metabolites, 3-hydroxy kynurenine and 3-hydroxy anthranilic acid (known antioxidants), in an NRF2-dependent, AHR independent manner [29,30]. Numerous studies have now implicated various aspects of cellular stress to changes in: GSH for Alzheimer’s [31], IBD [32,33], liver disease [34], changes in the pool of constituent amino acids [35], the glutathione assembly process and transport across cell membrane [36], or changes in the abundance of converting enzymes in ageing [8,37].

Here, we describe a novel method to quantitate the enzymes xCT, GSH1, GSH2, GPx, and GSHR, and the metabolites building blocks cysteine, cystine, and kynurenine as markers of oxidation. This multi-omics method, when applied to a singular sample, can provide an elegant and simple snapshot of the redox state from the same patient, same sample, and same time point for both the enzymes and metabolites. Thus, providing a holistic proteomic and metabolomic verification of cellular state and a potential biomarker panel that extends beyond the requirement for cellular (or tissue) extracts; with demonstrated utility across liquid biopsy samples of saliva, nasal swab, serums, and plasma.

## 2. Results and Discussion

Four sources of liquid biopsy were quantitated for targets of the glutathione redox metabolism pathway. Biomarkers of pathology are typically explored in serum and plasma because of their ability to provide real-time, non-invasive (compared to biopsy), and comprehensive insights from circulating proteins and metabolites that are specific to pathology or cellular dysfunction at concentrations amenable to analytical technologies. Saliva provides an even less invasive alternative to blood collection; however, care must be taken with consistent sampling to account for hypothalamic circadian fluctuations. Nasal swabs have also become accepted as a routine sampling method and provide a rich source of proteins and metabolites useful for the detection of disease related changes. Precision health evaluates individual fluctuations and measurements hold clinical significance for personalised medicine. Liquid biopsy is a form of diagnostic sampling allowing multi-omic approaches which are traditionally carried out in isolation of each other. Simultaneous extraction of lipids, metabolites, and proteins is possible [38]. However, the method is not often used with the foresight to explore the connections within a substrate type, nor between substrates for individual biochemical pathways. More often, there is emphasis placed on achieving the highest number of protein or metabolite identifications in proteomic and metabolomic studies, and this is at odds with the requisite for targeted, personalised medicine.

Here, we focus on the GSH pathway, which already has a significant body of research supporting its pivotal role in health; its critical role in redox balance; and emerging links to tryptophan metabolism, energy function, and inflammation. The strength of this multi-omic method stems from its simple extraction, adaptability to multiple liquid biopsy and cellular samples, and ‘universal equity’ to metabolomic and proteomic assessment without loss of either approach from the same patient at the same time point and under the same conditions of sample preparation. We demonstrate the application of the method to saliva, nasal swabs, plasma, and serum, and successful application in the context of a pathology-IBD.

### 2.1. Assessment of Biological Matrices for Targeted Protein Analysis

**Figure 2:**
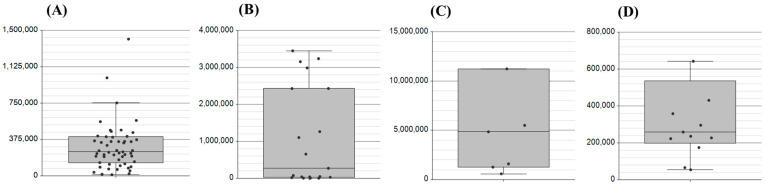
Evidence for the detection of the substrate anti-porter xCT (SLC7A) across the biological matrices of (**A**) saliva, (**B**) serum, (**C**) plasma, and (**D**) nasal swabs. Area under the peak (AUC) was assessed for the peptide KPVVSTISK with an MH^2+^ *m*/*z* of 479.8008.

Proteomic analysis of the GSH shunt is novel, and thus, our focus was to firstly establish protein targets with direct relationships to the shunt. We explored cellular access to the substrate building blocks by targeting the anti-porter xCT (SLC7A). Although a membrane protein, xCT detection in blood provides a good basis for assessment in liquid biopsy (https://www.proteinatlas.org; accessed on 4 April 2025). Here, we have been able to demonstrate xCT presence in saliva, nasal swabs, plasma and serum (Figure 2).

**Figure 3:**
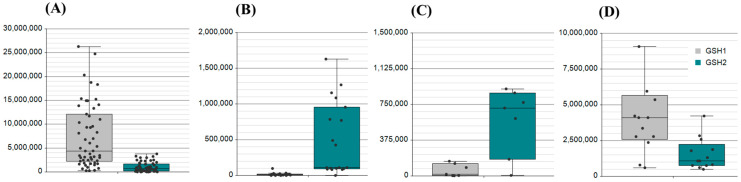
Evidence for the detection of the enzymes GSH1 and GSH2 across the biological matrices of (**A**) saliva, (**B**) serum, (**C**) plasma, and (**D**) nasal swabs. AUC was assessed for the GSH1 peptide DPLTLFEEK (Grey box) with an MH^2+^ *m*/*z* of 546.2846. Serum GSH1 abundances had a median AUC of 10,000. AUC was assessed for GSH2 peptide (Green box) AIENELLAR with an MH^2+^ *m*/*z* of 514.7909 in saliva, serum and plasma while ALAEGVLLR with an MH^2+^ *m*/*z* of 471.2926 was detected in nasal swabs.

The enzymes responsible for the generation of the GSH tripeptide GSH1 and GSH2 were also observed. However, uniquely nasal swabs, detection was based on signature transitions belonging to the peptide ALAEGVLLR, while saliva, plasma and serum relied on the tryptic peptide AIENELLAR (Figure 3).

**Figure 4:**
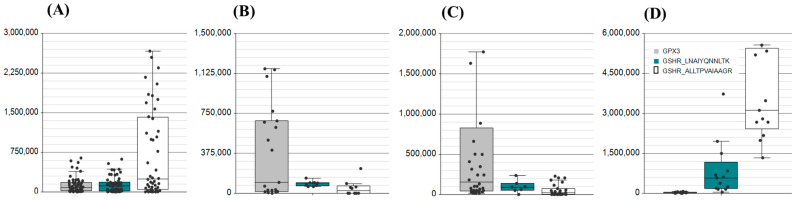
Evidence for the detection of the GSH peroxidase enzyme GPx3 and the reductase GSHR across the biological matrices of (**A**) saliva, (**B**) serum, (**C**) plasma, and (**D**) nasal swabs. AUC was assessed for the GPx3 peptide QEPGENSEILPTLK (Grey box) with an MH^2+^ *m*/*z* of 777.9041, GSHR peptides LNAIYQNNLTK (Green box) with an MH^2+^ *m*/*z* of 646.3539, and ALLTPVAIAAGR (white box) with an MH^2+^ *m*/*z* of 576.8586.

Redox homeostasis is maintained by two proteins and includes the conversion of rGSH to the oxidised form by GPx, and the regeneration of the reduced form by GSHR. These enzymes are detectable in all liquid biopsies and were also identified with extensive transitions and multiple peptide targets across all matrices (Figure 4).

### 2.2. Application of the Method to Samples of Pathological Significance in IBD

Classically, quite complex, invasive, and costly methods [4,20,39,40] have been used to measure the metabolites GSH and GSSG as health indicators independent of the complete redox control network, providing a restricted view of cellular redox. The ability to detect both the enzymes responsible for conversion of GSH to GSSG, as well as proteins responsible for GSH generation and transport using easily accessible liquid biopsy samples, using this universal method holds promise for the exploration of their potential as markers of health across numerous pathologies including cancer, chronic illnesses, and IBD.

**Figure 5:**
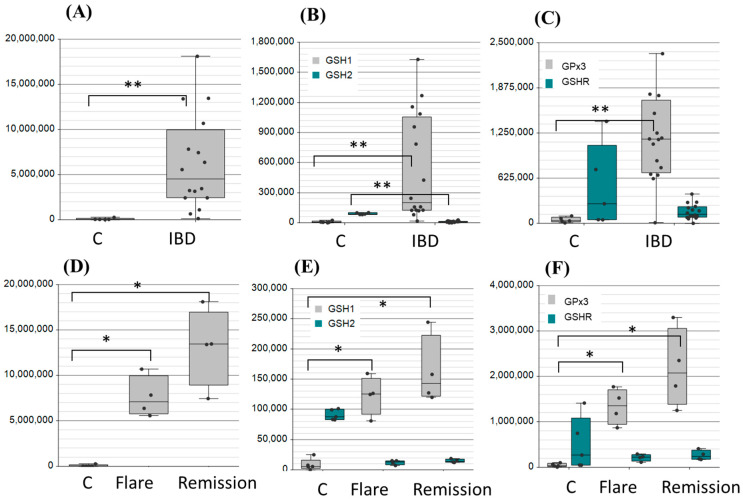
PRM targeted confirmation of protein markers of redox potential applied to IBD as a demonstrative pathological utility. (**A**) xCT (SLC7A); (**B**) GSH1 and GSH2; and (**C**) GPx3 and GSHR. Patient matched (*n* = 4) flare and remission data demonstrate a similar trend for (**D**) xCT, (**E**) GSH1 and GSH2, and (**F**) GSHR. Significance *p* < 0.05 represented by *; *p* < 0.005 represented by **.

**Figure 6:**
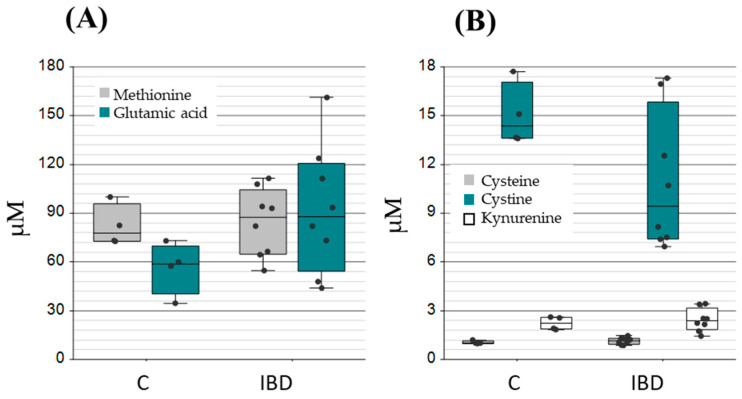
Metabolomic quantitation of substrate building blocks of the GSH shunt. (**A**) The amino acids methionine and glutamic acid, (**B**) the amino acid cysteine, cystine, and an important SLC7A anti-ported tryptophan metabolism product Kynurenine in an NRF2-dependent manner with links to ferroptosis. All measurements are in μM.

**Figure A1:**
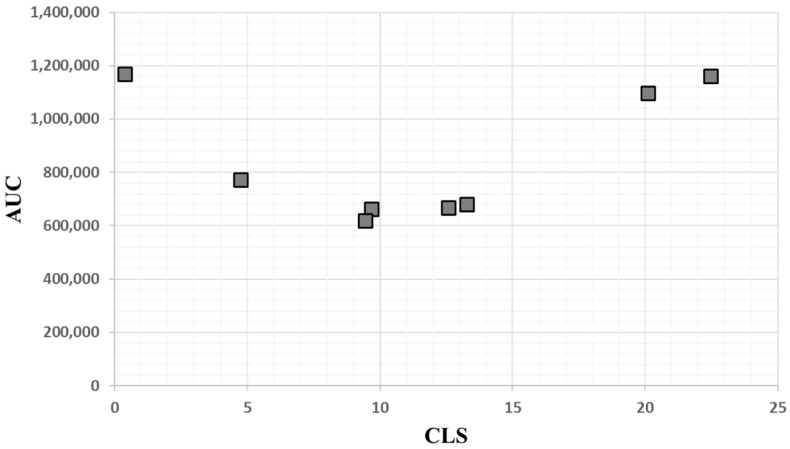
Relative GPx3 levels in patients categorised by confocal laser endoscopy scoring, showing an inflexion point at a CLS score of ~10. Definitions for this form of defining IBD by gut leakiness has previously been described [42].

The role of GSH in the management of oxidative stress in intestinal tissue injury related to IBD has shown the need for robust measures of redox state and cellular health [33,41,42]. The methods employed in these studies, while successful in demonstrating changes in glutathione levels in blood and biopsy samples, were invasive. Numerous studies rely on animal sacrifice to assess GSH and redox in the context of enteropathies and form hypotheses due to the invasive nature of these assessments. Many of the reported approaches of analysis, therefore, lack further clinical value. Here, we can demonstrate the application of our method to IBD pathology with metabolomic and proteomic evaluation of the GSH shunt. Figure 5 provides a snapshot of proteomic targeted analysis and metabolomic substrates quantification (Figure 6) in IBD. Significant differences were observed between non-IBD controls and clinically diagnosed IBD patients (significance notated as * < 0.05, ** < 0.005 in Figure 5). These differences were also pronounced in patient matched flare-up samples. Based on these observable differences, we considered modifications to the GPx/GSHR ratio as a diagnostic indicator of redox state. The protein ratio for IBD samples is 8-fold, favouring increased GPx, thus, driving the glutathione reaction towards consuming GSH and neutralizing peroxides. IBD patients, categorised by small intestinal permeability as detected by confocal laser endomicroscopy scoring (CLS), showed a divergent trend toward higher levels of GPx3 at the extremes of the CLS scale (Figure A1). No significant difference was observed between IBD patients and non-IBD controls in the metabolite substrates, conceivably showing compensatory mechanisms to maintain metabolite homeostasis.

Therapeutically, there may be a benefit to the treatment of cellular injury by ROS inundation with enzymes such as GPx or GSHR to encourage the detoxification of peroxides. This strategy has been tested in the context of hepatic ischaemia re-perfusion injury to reduce site-specific inflammation [8]. An ability to easily account for these proteins could therefore provide a more clinically acceptable approach to determining redox status with prognostic or diagnostic benefit into the future.

## 3. Materials and Methods

### 3.1. Sample Information

Serum, plasma, saliva, nasal swabs, or cellular extracts may be prepared using a parallel method optimal for protein and metabolites [1]. Here, we focus on liquid biopsy. Serum and plasma samples were collected by standard procedures as previously noted [1]. Saliva sampling was performed by oral swabs wiped along the inside of the cheek. Patient serum samples were categorized by pathology only and included paired inflammatory bowel disease with ‘flare’ versus non-‘flare’ patient matching (*n* = 4), and ranging in severity based on confocal laser endomicroscopy (CLS) [43] for 8 patients (ranging from CLS of 0.3–22.5). All other samples included healthy controls, chronic disease, and cancer to cover a broad range of marker abundances, with the main goal of this research aimed at establishing markers of the glutathione shunt. These samples were used as a demonstration of the method as well as the functionality across many biological matrices and are described in Table 1.

Adaptions from a simple 80% methanol protein crash can be considered [1] for proteomic analysis only. However, GSH is not soluble in alcohol and thus requires aqueous TCA for extraction. Quantities of GSH and GSSG in these sample types were below our detection limits; however, the method would be adaptable for tissue or cell extract where these metabolites are in higher quantities. The metabolites are also detectable using N-Ethylmaleimide (NEM) derivatisation, but the method cannot be run concurrently with other amino acid quantifications used here. For in-parallel multi-omic analysis, a final 2% TCA protein precipitation was used to re-suspend the samples. Following TCA precipitation and centrifugation for 30 min at 14,000× *g*, the supernatant is reserved for the metabolomics arm of analysis, while the protein pellet is reserved for the proteomic arm of analysis. The protein pellet was washed with 200 µL 80% methanol (−20 °C for 1 h) and centrifuged to collect pellets for 5 min at 14,000× *g* [44].

### 3.2. Targeted Protein Analysis by Parallel Rection Monitoring (PRM)

A total of 100 μg of protein was re-suspended in 100 µL AMBIC, 10 mM DTT, 2 M urea at pH 8, was used for trypsin digestion at 25 °C for 16 h in a 1:100 ratio. Sample clean-up was performed using C18 Stage tips as previously described [1]. Proteomic analysis was conducted using a Vanquish Neo Nano UPLC system and an Orbi-trap Exploris 480 mass spectrometer (Thermo Fisher Scientific, Waltham, MA, USA) equipped with a high-field asymmetric waveform ion mobility spectrometer (FAIMS).

PRM followed specific parent ions and transition ions of marker proteins provided in Table A1. Injection was 0.7 µL from 10 µL for saliva samples, 1 µL from 10 µL for serum and plasma samples, and 1 µL from 10 µL for nasal swab. The total ion current for each sample was used to normalise using relative quantitative techniques. A strict elution window of +/−2 min and at least 4 transitions were used, when possible, to limit any erroneous identifications. Peptides were eluted using an in-house manufactured 24 cm, 75 μm i.d., C18 column (1.9 μm, 120 Å, Dr. Maisch HPLC GmbH, Ammerbuch, Germany) using a linear gradient of H_2_O:CH_3_CN (98:2, 0.1% formic acid) to H_2_O:CH_3_CN (20:80, 0.1% formic acid) at 200 nL/min over 80 min.

**Figure A2:**
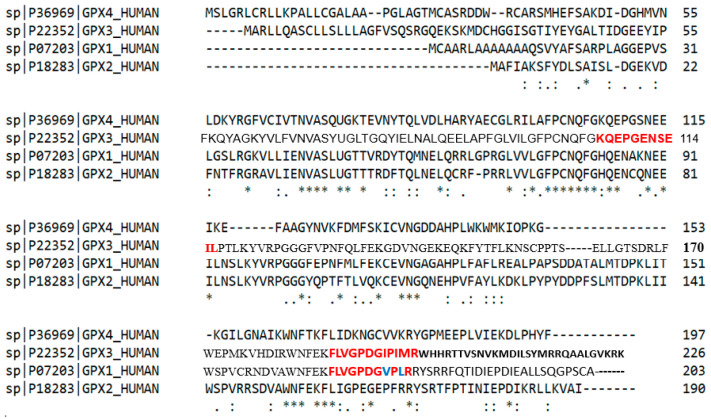
Multiple sequence alignment of the GPx. Highlighted sequences indicate unique peptides. Indications of identity are represented by *, similarity are represented by dots, gaps in the identity are represented by dash.

The MS was operated in positive ion mode with a voltage of 2000 V and a full scan at 30,000 resolution within a mass window of 350–1750 *m*/*z* in profile mode. FAIMS was run with standard resolution, with CV set to −45 V to select ≥2–6 charge states. Peak width was set to 23 s with an isolation window of 1.5 Da. The full scan was collected with the maximum injection time set to auto and MS2 was collected in centroid mode. Unique peptide transitions were developed in the Skyline environment [45] using the software’s default settings. Data files were imported for manual inspection and interference assessment. The peak area under curve of the parent ion was used to assess relative abundance of the targets. As several isozymes encoded by different genes are apparent for Glutathione Peroxidase, further targets beyond GPx3 were added for future work involving intracellular extracts and analysed in liquid biopsy. Multiple sequence alignment was used to confirm the specificity of GPx enzymes in human patients using Clustal Omega via EMBL version 1.2.4 (https://www.ebi.ac.uk/Tools/msa/clustalo/, accessed on 8 April 2025) [46] (See Figure A2).

### 3.3. Targeted Metabolite Analysis

Metabolites were measured using a liquid chromatography–tandem mass spectrometry (LC–MS/MS) approach using a TSQ Vantage mass spectrometer (Thermo Fisher Scientific, Waltham, MA, USA) fitted with a heated electrospray probe (HESI) coupled to an integrated Vanquish pump/autosampler system.

Chromatographic separation was achieved using a Kinetex™PFP column (150 mm, 2 mm, 3 μm, 100 Å, Phenomenex, Torrance, CA, USA). Reversed phase buffers consisted of 100% water acidified with 0.1% formic acid (mobile phase A) and methanol (mobile phase B). Initial conditions were (flow rate 125 μL/min) 100% A, held for two minutes, ramped to 20% B in one minute, then increased to 70% B at 5 min. The metabolites of interest were eluted in this time frame. The column was washed at 100% B for 8 min (flow rate increased to 250 μL/min) and equilibrated for 6 min before the next injection; this was crucial to maintain peak shape.

All MS/MS transitions were acquired in positive polarity mode, and those with the best signal intensity and less interference were selected. Requisite transitions are detailed in Table A2. Mass spectra were accumulated during 0.25 s per SRM. MSD capillary voltage, capillary temperature, and collision gas pressure (Argon) were set to 5000 V, 300 °C, and 1.0 Torr, respectively. Sheath and auxiliary gas valves (nitrogen) were set at 10 and 5 arbitrary units.

**Figure A3:**
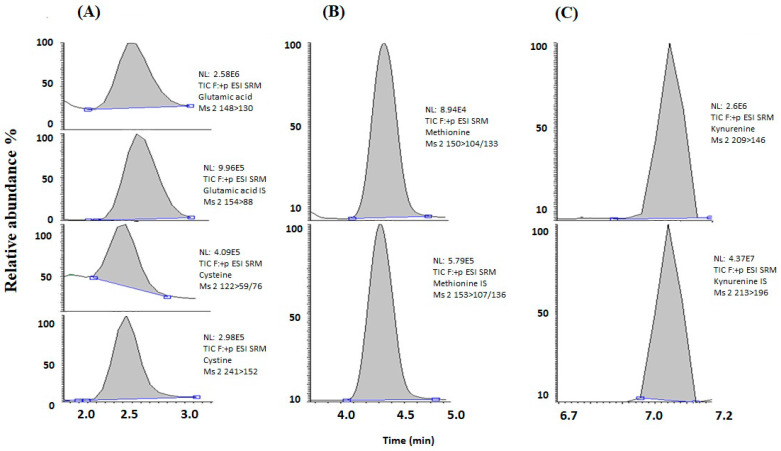
Mass chromatogram of serum sample (8-fold dilution) precipitated with TCA using a 20 min gradient. (**A**) shows Glutamic acid (*m*/*z* 148) and labelled glutamate (*m*/*z* 154). The metabolites cysteine (*m*/*z* 122) and cystine (*m*/*z* 241) were quantitated using labelled glutamate, which elutes at similar retention time. (**B**) shows methionine (*m*/*z* 150) and labelled methionine (*m*/*z* 153); (**C**) shows kynurenine (*m*/*z* 209) and labelled kynurenine (*m*/*z* 213).

Calibration curves for each individual amino acid were plotted using the peak area ratios (peak area of the metabolite divided by the peak area of its allocated internal standard; Y axis) versus calibrator concentration (Figure A3). Peak areas were integrated using Xcalibur™ software (version 2.2, 2011, Thermo-Fisher Scientific, Waltham, MA, USA). The concentrations of the endogenous metabolites in the serum TCA extracts were obtained from these calibration curves, accounting for dilution factors.

### 3.4. Statistical Analysis

Response and group variables were used to calculate significance using a two-sample *t*-test. This study can provide benefits by demonstrating method utility in a real-world scenario. Tests between control and IBD serums achieved 85% power. Power was calculated using a two-sample *t*-test assuming equal variance in NCSS (v9.0.5) and PASS (v12.0.3) software environment.

## 4. Conclusions

This multi-omics method encompasses both the intracellular spill out and the extra cellular availability of metabolites and proteins in readily available liquid biopsies. Our study has been successful in demonstrating utility across a broad range of biological matrices and was applied to a disease scenario. Recognising the differences in the protein ratio and metabolite ratio to uniquely contribute to measures of redox homeostasis is an important question that will require further evaluation. Further studies with increased power will decipher the ability of these markers to differentiate oxidative stress in disease and health. The approach integrating proteomics and related metabolites can be used to enhance understanding of GSH-related redox balance and has the potential to provide mechanistic insights and identify compensatory responses for oxidative-stress related diseases such as cancer, chronic diseases, and diseases of ageing. Proteins and metabolites can act as complementary biomarkers, enhancing accuracy in disease detection and/or monitoring. This may provide a valuable clinical measurement for disease monitoring of redox status, with the possibility of simple pharmaceutical treatment options already available such as GSH, amino acid precursors, or potentially enzymes into the future.

## Figures and Tables

**Table 1:** Adult samples analysed, matrices, and method of application.

Matrix.	Count	Application: Metabolomic (M), Proteomic (P)
Saliva	50	P
Plasma	25	M, P
Serum	25 (16 IBD)	M, P
Nasal swab	12	P

## Data Availability

All details required to reproduce this method are provided in the body of the paper and Appendix A.

## Abbreviations

The following abbreviations are used in this manuscript:

rGSH	reduced glutathione
GSSG	oxidised glutathione
PRM	parallel reaction monitoring
SRM	single reaction monitoring

## Appendix A

**Table A1:** PRM transitions monitored for proteins associated with the GSH shunt.

Protein	ID	Sequence	Parent *m*/*z*	z	Product *m*/*z*	Peak
GPx3 *	P22352	K.QEPGENSEILPTLK	777.9041	2	1200.6470 1014.5830 900.5401 458.2973 649.3535	
		K.FLVGPDGIPIMR	657.8656	2	1054.5714 955.5030 898.4815 801.4287 686.4018 516.2963	
GSHR	P00390	R.LNAIYQNNLTK ^#^	646.3539	2	1178.6164 1064.5735 993.5364 717.3890	
		K.ALLTPVAIAAGR	576.8586	2	968.5887 855.5047 754.4570 657.4042 377.7321 399.2602	
SLCA7/xCT	Q9UPY5	R.KPVVSTISK	479.8008	2	830.4982 415.7527 129.1022	
GSH2	P48637	R.AIENELLAR	514.7909	2	557.2566 670.3406 783.4247	
		R.ALAEGVLLR ^#^	471.2926	2	757.4567 686.4196 557.3770 500.3555	
GSH1	P48506	R.DPLTLFEEK	546.2846	2	879.4822 766.3981 665.3505	

* GPx: targets share common peptides. GPx3 is predominantly present in plasma and known as extracellular glutathione peroxidase. GPx1, 2, 4 are intracellular peroxidase [47]. Human GPx1, 2, 4 peptides were not observed, however GPx4 is detectable in animal serum (unpublished data). ^#^ Observed in saliva and nasal swab.

**Table A2:** SRM transitions monitored for substrates associated with the GSH shunt.

Metabolites	Retention Time	Precursor Ion (*m*/*z*)	Product Ions (*m*/*z*)	Collision Energy (eV)
Glutamic acid	2.45	148	130/84	20
^13^C ^15^N-Glutamic acid	2.48	154	89	20
Cysteine	2.30	122	59/76	12
Cystine	2.34	241	152	20
Methionine	4.33	150	104/133	12
^2^H_3_-methionine	4.32	153	107/136	12
Kynurenine	7.05	209	146	20
^2^H_4_-kynurenine	7.04	213	196	12
